# Dataset for the NMR structure of the intrinsically disordered acidic region of XPC bound to the PH domain of TFIIH p62

**DOI:** 10.1016/j.dib.2015.12.034

**Published:** 2016-01-02

**Authors:** Masahiko Okuda, Yoshifumi Nishimura

**Affiliations:** Graduate School of Medical Life Science, Yokohama City University, 1-7-29 Suehiro-cho, Tsurumi-ku, Yokohama 230-0045, Japan

## Abstract

The global genome nucleotide excision repair factor XPC firstly detects DNA lesions and then recruits a ten-subunit complex TFIIH through binding to the subunit p62 to unwind the damaged DNA for excision repair. This data article contains detailed nuclear magnetic resonance (NMR) restraints (nuclear Overhauser enhancement (NOE)-derived distance restraints, dihedral angle restraints, and hydrogen bond restraints) used for the structure determination of the complex formed between the intrinsically disordered acidic region of XPC and the pleckstrin homology (PH) domain of TFIIH p62, related to the recent work entitled “Structural insight into the mechanism of TFIIH recognition by the acidic string of the nucleotide excision repair factor XPC.” [1].

## **Specifications Table**

**Table:** 

Subject area	Structural biology
More specific subject area	Nuclear magnetic resonance, NMR
Type of data	NMR restraints, table, figure
How data was acquired	Solution NMR
Data format	Analyzed
Experimental factors	No sample pretreatment applied
Experimental features	NMR samples were 170–190 μl of 400 μM protein complex (^13^C,^15^N-protein: unlabeled protein=1.0: 1.2 M ratio) solution in 20 mM potassium phosphate (pH 6.8), 5 mM deuterated DTT, and either 10.0% D_2_O or 99.9% D_2_O; All data was acquired at 305 K.
Data source location	Yokohama City University, Yokohama, Japan
Data accessibility	Data is provided as Supplementary material directly with this article.
The structural coordinates have been deposited to RCSB Protein Data Bank (http://www.rcsb.org) (PDB: 2RVB).

## **Value of the data**

• The dataset helps researchers to design their NMR experiments.
• The dataset is useful for the trial calculation of a protein complex structure.
• The detailed NMR restraint dataset is useful for evaluation of structure simulation procedures of a protein complex by using a limited amount of data from the data set.
• The dataset provides structural insights into intrinsically disordered regions.

## 1 Data

We prepared the XPC fragment (residues 109–156) and the p62 PH domain (residues 1–108) from *Escherichia coli* expression systems [1], [2]. The XPC fragment contains an intrinsically disordered acidic region (residues 124–141), which forms an elongated string-like structure upon binding to the p62 PH domain [1]. We used four samples for the structure determination by NMR, namely:

(a) complex of 400 μM ^13^C,^15^N-labeled XPC with 480 μM unlabeled p62 PH domain in 10.0% D_2_O (XPC-p62_H_2_O),
(b) complex of 400 μM ^13^C,^15^N-labeled XPC with 480 μM unlabeled p62 PH domain in 99.9% D_2_O (XPC-p62_D_2_O),
(c) complex of 400 μM ^13^C,^15^N-labeled p62 PH domain with 480 μM unlabeled XPC in 10.0% D_2_O (p62-XPC_H_2_O), and
(d) complex of 400 μM ^13^C,^15^N-labeled p62 PH domain with 480 μM unlabeled XPC in 99.9% D_2_O (p62-XPC_D_2_O).

NMR data were acquired on Bruker AVANCE III HD 600 MHz, AVANCE III HD 700 MHz, and AVANCE III HD 800 MHz spectrometers, each equipped with a cryogenic probe. NMR experiments used are summarized in Table 1.

## 2 Experimental design, materials and methods

### 2.1 NOE-derived distance restraints

In total, 182 and 2545 NOE-derived distance restraints were obtained for, respectively, XPC_109–156_ and TFIIH p62 PH domain (Table 2) [1].

#### 2.1.1 Distance restraints from the intramolecular NOEs

For XPC_109-156_ in complex, 73 NOEs (7 intraresidue NOEs; 48 sequential NOEs; 18 medium-range NOEs; 0 long-range NOE) were obtained from the ^13^C-edited NOESY-HSQC (mixing time (*τ*_m_) 100 ms) using the sample of XPC-p62_D_2_O (Table S1) and 109 NOEs (10 intraresidue NOEs; 86 sequential NOEs; 13 medium-range NOEs; 0 long-range NOE) were obtained from the ^15^N-edited NOESY-HSQC (*τ*_m_, 150 ms) using the sample of XPC-p62_H_2_O (Table S2). In the ^13^C-edited NOESY-HSQC we used *τ*_m_ of 100 ms, shorter than *τ*_m_ of 150 ms used in the ^15^N-edited NOESY-HSQC to avoid spin-diffusion problems.

For the p62 PH domain in complex, 1367 NOEs (171 intraresidue NOEs; 204 sequential NOEs; 233 medium-range NOEs; 759 long-range NOEs) were obtained from the ^13^C-edited NOESY-HSQC (*τ*_m_, 100 ms) using the sample of p62-XPC_D_2_O (Table S3) and 1178 NOEs (197 intraresidue NOEs; 419 sequential NOEs; 223 medium-range NOEs; 339 long-range NOEs) were obtained from the ^15^N-edited NOESY-HSQC (*τ*_m_, 150 ms) using the sample of p62-XPC_H_2_O (Table S4).

Note that we chose the intraresidue NOEs from only residues whose side-chains were stereo-specifically assigned.

#### 2.1.2 Distance restraints from the intermolecular NOEs

**Fig. 1:**
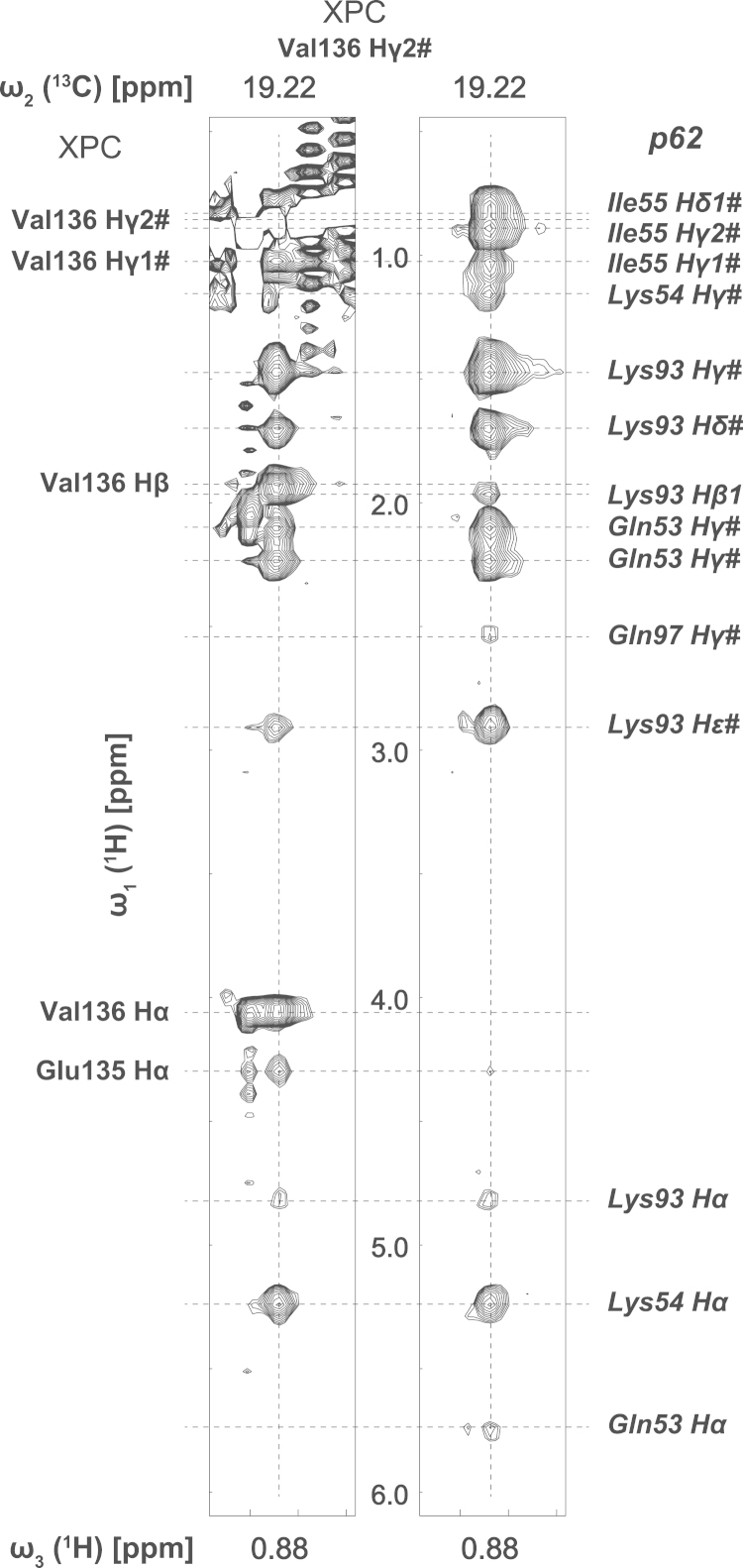
Intermolecular NOEs between the ^13^C,^15^N labeled XPC_109–156_ and the unlabeled p62 PH domain. Left: the strip of Val136 Hγ2 of XPC extracted from the ^13^C-edited NOESY-HSQC spectra. Right: the strip from the ^13^C,^15^N-filtered, ^13^C-edited NOESY-HSQC spectra.

The ^13^C,^15^N-filtered, ^13^C-edited NOESY-HSQC (*τ*_m_, 120 ms) for the sample of XPC-p62_D_2_O provided 162 intermolecular NOEs (Fig. 1). The ^13^C,^15^N-filtered, ^15^N-edited NOESY-HSQC (*τ*_m_, 150 ms) for the sample of XPC-p62_H_2_O provided 54 intermolecular NOEs.

The ^13^C,^15^N-filtered, ^13^C-edited NOESY-HSQC (*τ*_m_, 120 ms) for the sample of p62-XPC_D_2_O provided 107 intermolecular NOEs. The ^13^C,^15^N-filtered, ^15^N-edited NOESY-HSQC (*τ*_m_, 150 ms) for the sample of p62-XPC_H_2_O provided 49 intermolecular NOEs.

Removing duplicated restraints, we acquired 199 intermolecular NOEs from the ^13^C-edited NOESY (Table S5) and 100 intermolecular NOEs from the ^15^N-edited NOESY (Table S6).

### 2.2 Dihedral angle restraints

The analysis of the backbone chemical shift (^15^N, ^13^Cα, ^13^Cβ, ^13^C′, and Hα) with TALOS+ [4] generated 9 φ and 9 ψ for XPC_109-156_ in complex (Tables 2 and S7), and 96 φ and 95 ψ for the p62 PH domain in complex (Tables 2 and S8).

The side-chain torsion angles were analyzed by the HNHB, HN(CO)HB, HNCG, HN(CO)CG, ^13^C-edited NOESY-HSQC (*τ*_m_, 50 ms) and ^15^N-edited NOESY-HSQC (*τ*_m_, 50 ms), and 3 χ1 for XPC_109-156_ in complex (Tables 2 and S7) and 63 χ1, 10 χ2 for the p62 PH domain in complex were determined (Tables 2 and S8).

### 2.3 Hydrogen bond restraints

We performed the H/D-exchange experiment to obtain hydrogen bond restraints. As a reference spectrum, a ^1^H, ^15^N HSQC spectrum was taken for the sample of p62-XPC_H_2_O. We prepared the lyophilized sample of p62-XPC_H_2_O, and then immediately after adding D_2_O to the lyophilized sample, a series of ^1^H,^15^N HSQC spectra were taken. Hydrogen-bond donors were identified by comparing those spectra with the reference spectrum. Hydrogen-bond donor–acceptor pairs were determined based on the final structure.

The H/D-exchange experiment provided 96 (48×2) hydrogen bond restraints for the p62 PH domain in complex (Tables 2 and S9). No hydrogen bond restraints were available for XPC_109-156_, because of the fast H/D-exchange.

## Figures and Tables

**Table 1:** NMR experiments used for the structure determination.

^13^C,^15^N-XPC/unlabeled p62	^13^C,^15^N-p62/unlabeled XPC
**Backbone assignment**	
CBCANH^a^	CBCANH^b^
[6.6(*t*_1_,^13^C), 14.5(*t*_2_,^15^N), 148.7(*t*_3_,^1^H_N_)]^c^	[6.6(*t*_1_,^13^C), 9.9(*t*_2_,^15^N), 148.7(*t*_3_,^1^H_N_)]^c^
CBCA(CO)NH^a^	CBCA(CO)NH^b^
[6.6(*t*_1_,^13^C), 14.5(*t*_2_,^15^N), 148.7(*t*_3_,^1^H_N_)]^c^	[6.6(*t*_1_,^13^C), 9.9(*t*_2_,^15^N), 148.7(*t*_3_,^1^H_N_)]^c^
HNCA^a^	
[20.2(*t*_1_,^13^C), 15.5(*t*_2_,^15^N), 148.7(*t*_3_,^1^H_N_)]^c^	
HN(CO)CA^a^	
[20.2(*t*_1_,^13^C), 15.5(*t*_2_,^15^N), 148.7(*t*_3_,^1^H_N_)]^c^	
HN(CA)CO^a^	HN(CA)CO^b^
[18.3(*t*_1_,^13^C), 15.5(*t*_2_,^15^N), 148.7(*t*_3_,^1^H_N_)]^c^	[18.3(*t*_1_,^13^C), 10.5(*t*_2_,^15^N), 148.7(*t*_3_,^1^H_N_)]^c^
HNCO^a^	HNCO^b^
[18.3(*t*_1_,^13^C), 15.5(*t*_2_,^15^N), 148.7(*t*_3_,^1^H_N_)]^c^	[18.3(*t*_1_,^13^C), 10.5(*t*_2_,^15^N), 148.7(*t*_3_,^1^H_N_)]^c^

**Side-chain assignment**	
HBHANH^a^	
[8.7(*t*_1_,^1^H), 14.5(*t*_2_,^15^N), 148.7(*t*_3_,^1^H_N_)]^c^	
HBHA(CO)NH^a^	HBHA(CO)NH^b^
[8.7(*t*_1_,^1^H), 14.5(*t*_2_,^15^N), 148.7(*t*_3_,^1^H_N_)]^c^	[8.7(*t*_1_,^1^H), 9.9(*t*_2_,^15^N), 148.7(*t*_3_,^1^H_N_)]^c^
HCCCONH^a^	
[9.3(*t*_1_,^1^H), 13.5(*t*_2_,^15^N), 148.7(*t*_3_,^1^H_N_)]^c^	
CCCONH^a^	
[5.4(*t*_1_,^13^C), 15.5(*t*_2_,^15^N), 148.7(*t*_3_,^1^H_N_)]^c^	
HCCH-TOCSY^d^	HCCH-TOCSY^e^
[12.2(*t*_1_,^1^H), 2.9(*t*_2_,^13^C), 174.1(*t*_3_,^1^H)]^c^	[12.2(*t*_1_,^1^H), 2.8(*t*_2_,^13^C), 174.1(*t*_3_,^1^H)]^c^
HCCH-COSY^d^	HCCH-COSY^e^
[12.2(*t*_1_,^1^H), 2.9(*t*_2_,^13^C), 174.1(*t*_3_,^1^H)]^c^	[12.2(*t*_1_,^1^H), 2.8(*t*_2_,^13^C), 174.1(*t*_3_,^1^H)]^c^

**Stereo-specific assignment**	
HNHB^a^	HNHB^b^
[7.1(*t*_1_,^1^H), 14.5(*t*_2_,^15^N), 148.7(*t*_3_,^1^H_N_)]^c^	[7.1(*t*_1_,^1^H), 9.8(*t*_2_,^15^N), 148.7(*t*_3_,^1^H_N_)]^c^
HN(CO)HB^a^	HN(CO)HB^b^
[7.1(*t*_1_,^1^H), 14.5(*t*_2_,^15^N), 148.7(*t*_3_,^1^H_N_)]^c^	[7.1(*t*_1_,^1^H), 9.8(*t*_2_,^15^N), 148.7(*t*_3_,^1^H_N_)]^c^
HNCG^a^	HNCG^b^
[5.2(*t*_1_,^13^C), 14.5(*t*_2_,^15^N), 148.7(*t*_3_,^1^H_N_)]^c^	[5.2(*t*_1_,^13^C), 9.8(*t*_2_,^15^N), 148.7(*t*_3_,^1^H_N_)]^c^
HN(CO)CG^a^	HN(CO)CG^b^
[5.2(*t*_1_,^13^C), 14.5(*t*_2_,^15^N), 148.7(*t*_3_,^1^H_N_)]^c^	[5.2(*t*_1_,^13^C), 9.8(*t*_2_,^15^N), 148.7(*t*_3_,^1^H_N_)]^c^^13^C NOESY-HSQC (*τ*_m_, 50 ms)^e^
^13^C NOESY-HSQC (*τ*_m_, 50 ms)^d^	
[13.1(*t*_1_,^1^H), 2.6(*t*_2_,^13^C), 111.4(*t*_3_,^1^H)]^c^	[13.1(*t*_1_,^1^H), 3.0(*t*_2_,^13^C), 111.4(*t*_3_,^1^H)]^c^
^15^N NOESY-HSQC (*τ*_m_, 50 ms)^a^	^15^N NOESY-HSQC (*τ*_m_, 50 ms)^b^
[7.0(*t*_1_,^1^H), 11.6(*t*_2_,^15^N), 111.4(*t*_3_,^1^H_N_)]^c^	[7.0(*t*_1_,^1^H), 11.3(*t*_2_,^15^N), 111.4(*t*_3_,^1^H_N_)]^c^

**Distance restraints**	
^13^C NOESY-HSQC (*τ*_m_, 100 ms)^d^	^13^C NOESY-HSQC (*τ*_m_, 100 ms)^e^
[13.1(*t*_1_,^1^H), 3.0(*t*_2_,^13^C), 111.4(*t*_3_,^1^H)]^c^	[13.1(*t*_1_,^1^H), 3.0(*t*_2_,^13^C), 111.4(*t*_3_,^1^H)]^c^
^15^N NOESY-HSQC (*τ*_m_, 150 ms)^a^	^15^N NOESY-HSQC (*τ*_m_, 150 ms)^b^
[7.0(*t*_1_,^1^H), 11.6(*t*_2_,^15^N), 111.4(*t*_3_,^1^H_N_)]^c^^13^C,^15^N-filtered ^13^C-edited	[7.0(*t*_1_,^1^H), 11.3(*t*_2_,^15^N), 111.4(*t*_3_,^1^H_N_)]^c^^13^C,^15^N-filtered ^13^C-edited
NOESY-HSQC (*τ*_m_, 120 ms)^d^	NOESY-HSQC (*τ*_m_, 120 ms)^e^
[12.0(*t*_1_,^1^H), 3.0(*t*_2_,^13^C), 111.4(*t*_3_,^1^H)]^c^	[11.6(*t*_1_,^1^H), 3.0(*t*_2_,^13^C), 111.4(*t*_3_,^1^H)]^c^
^13^C,^15^N-filtered ^15^N-edited NOESY-HSQC (*τ*_m_, 150 ms)^a^	^13^C,^15^N-filtered ^15^N-edited NOESY-HSQC (*τ*_m_, 150 ms)^b^
[7.0(*t*_1_,^1^H), 11.6(*t*_2_,^15^N), 111.4(*t*_3_,^1^H_N_)]^c^	[7.0(*t*_1_,^1^H), 11.3(*t*_2_,^15^N), 111.4(*t*_3_,^1^H_N_)]^c^

**Dihedral restraints**	
(φ,ψ): Backbone assignment	(φ,ψ): Backbone assignment
(χ_1_,χ_2_): Stereo-specific assignment	(χ_1_,χ_2_): Stereo-specific assignment

**Hydrogen bond restraints**	
^15^N-HSQC (H–D exchange)	^15^N-HSQC (H–D exchange)
[22.5(*t*_1_,^15^N), 111.4(*t*_2_,^1^H_N_)]^c^	[22.5(*t*_1_,^15^N), 111.4(*t*_2_,^1^H_N_)]^c^

a Sample of XPC-p62_H_2_O.

b Sample of p62-XPC_H_2_O.

c Maximum evolution times used in each dimension (ms).

d Sample of XPC-p62_D_2_O.

e Sample of p62-XPC_D_2_O.

**Table 2:** NMR restraints used for the structure determination.

	XPC	p62
**Distance restraints**^a^		
Intramolecular NOEs		
^13^C-edited NOESY-HSQC (*τ*_m_, 100 ms)	73^b^	1367^c^
Intraresidue (*i*–*j*=0)	7^b^	171^c^
Sequential (*i*–*j*=1)	48^b^	204^c^
Medium-range (1<*i*–*j*<5)	18^b^	233^c^
Long-range (*i*–*j*≥5)	0^b^	759^c^
		
^15^N-edited NOESY-HSQC (*τ*_m_, 150 ms)	109^d^	1178^e^
Intraresidue (*i*–*j*=0)	10^d^	197^e^
Sequential (*i*–*j*=1)	86^d^	419^e^
Medium-range (1<*i*–*j*<5)	13^d^	223 e
Long-range (*i*–*j*≥5)	0^d^	339^e^
		
Intermolecular NOEs	216	156
^13^C,^15^N-filtered ^13^C-edited NOESY-HSQC (*τ*_m_, 120 ms)	162	107
^13^C,^15^N-filtered ^15^N-edited NOESY-HSQC (*τ*_m,_ 150 ms)	54	49
^13^C,^15^N-filtered ^13^C-edited NOESY-HSQC (*τ*_m_, 120 ms)	199^f^	
^13^C,^15^N-filtered ^15^N-edited NOESY-HSQC (*τ*_m_, 150 ms)	100^g^	

**Dihedral restraints**		
Φ	9^h^	96^i^
Ψ	9^h^	95^i^
χ_1_	3^h^	63^i^
χ_2_	0^h^	10^i^
		
**Hydrogen bond restraints**		
	0	96 (48×2)^j^

a Distance restraints were obtained from analyses of NOE intensities by using NMRView [3].

b Supplementary Table S1.

c Supplementary Table S3.

d Supplementary Table S2.

e Supplementary Table S4.

f Supplementary Table S5.

g Supplementary Table S6.

h Supplementary Table S7.

i Supplementary Table S8.

j Supplementary Table S9.
