# Supplementary material for: Dataset for the NMR structure of the intrinsically disordered acidic region of XPC bound to the PH domain of TFIIH p62
Source: Data Brief. 2016 Jan 2;6:571–7. doi: 10.1016/j.dib.2015.12.034 (PMC4731421; doi:10.1016/j.dib.2015.12.034)
Supplement: Supplementary file 1 — Supplementary material [file mmc1.pdf]

December 4, 2015

Manuscript No.: DIB-D-15-00445
Title: Dataset for the NMR structure of the intrinsically disordered acidic region of XPC bound to the PH domain of TFIIH p62
Journal Title: Data in Brief
Corresponding Author: Prof. Yoshifumi Nishimura
All Authors: Masahiko Okuda, Ph.D.; Yoshifumi Nishimura, Ph.D.

Dear Dr. Hao-Ran Wang,

Thank you for your kind editorial effort on our manuscript with your voucher number, **DIB-2015-6**.

Here, I would like to submit our revised manuscript. In the revised version, we addressed almost all concerns raised by the reviewers as shown in a separate document addressing the points one by one.

In addition, I send you Conflicts of Interest form here.

We would greatly appreciate if you could consider this manuscript for publication in your journal.

Sincerely yours.

Yoshifumi Nishimura
Professor
Graduate School of Medical Life Science
Yokohama City University
1-7-29 Suehiro-cho, Tsurumi-ku, Yokohama 230-0045, Japan
Tel: 81-45-508-7211
Fax: 81-45-508-7360
e-mail: nisimura@tsurumi.yokohama-cu.ac.jp

We wish to confirm that there are no known conflicts of interest associated with this publication and there has been no significant financial support for this work that could have influenced its outcome.

We confirm that the manuscript has been read and approved by all named authors and that there are no other persons who satisfied the criteria for authorship but are not listed. We further confirm that the order of authors listed in the manuscript has been approved by all of us.

We confirm that we have given due consideration to the protection of intellectual property associated with this work and that there are no impediments to publication, including the timing of publication, with respect to intellectual property. In so doing we confirm that we have followed the regulations of our institutions concerning intellectual property.

We understand that the Corresponding Author is the sole contact for the Editorial process (including Editorial Manager and direct communications with the office). He is responsible for communicating with the other authors about progress, submissions of revisions and final approval of proofs. We confirm that we have provided a current, correct email address which is accessible by the Corresponding Author and which has been configured to accept email from nisimura@tsurumi.yokohama-cu.ac.jp

Signed by all authors as follows:

Masahiko Okuda

Masahiko Okuda December 4, 2015

Yoshifumi Nishimura

Y. Nishimura December 4, 2015
